# Neuromuscular Block and Video Laryngoscope to Facilitate Intubation—A Survey of Current Practice in Denmark and Sweden

**DOI:** 10.1111/aas.70200

**Published:** 2026-03-13

**Authors:** Åse Lodenius, Louise Holland‐Bill, Emil Ørskov Ipsen, Maria Cronhjort, Arash Afshari, Matias Vested, Andreas Creutzburg, Lars Hyldborg Lundstrøm, Helene Korvenius Nedergaard, Anders Kehlet Nørskov, ROCVIDEO Survey Collaborator Group, Mette Schytt Price, Christian Jessen, Mathias Sinkbæk Thomsen, Mikkel Schjødt Heide Jensen, Torben Krabbe Laustrup, Karina Baekby Houborg, Fabian Spies, Line Agger Kolstrup, Anni Nørgaard Jeppesen, Hanna Poulsen, Jannie Bisgaard, Hansjörg Selter, Cecilie Harries Klementsen, Hayan El‐Hallak, Daniel Hägi‐Pedersen, Jakob Hessel Andersen, Anders Lolle, Jacqueline Møller Mistry, Peter Martin Hansen, Kirsten Lerche Licht, Wojciech Jan Pietrzyk, Michael Leth Jørgensen, Christina Borgen, Anders Karlsen, Karsten Kindberg, Kim Wildgaard, Laila Mulla Reich, Simon Strøyer, Robin Lohse, Mette Lea Mortensen, Rune Lundsgaard, Mark Søgaard Niegsch, Erik Wikström, Anna Bandert, Carl Olsson, Malin Bohlin, Mälarsjukhuset Eskilstuna, Nanny Hareide, Olof Brattström, Karin Bursell, Lars Mattsson, Pontus Weinar, Katrin Ögren, Åsa Eriksson Nordesjö, Björn Ahlström, Petter Huitfeldt, Jakob Boethius, Tomi Myrberg, Jakob Walldén, Ulf A. Karlsson, Simon Näverlo, Jonas Rudenstam, Sofia Hannu, Pauline Koch, Thorey Steinarsdóttir, Gustaf Rosin, Anna Borglund Hemph, Jacob Broms, Liselott Wickerts, Anna Wennmo, Hanna Smeds, Mark Larsson, Jelena Paegle, Jonas Graf, Pierre Sundin, Katalin Rockstroh, Sebastian Felixson, Jim Runesson, Johan Berkius, Helena Krook, Louise Walther Sturesson, Fredrik Sjövall, Nikolaus Groll, Olof Nilsson, Johanna Broman, Susanne Gustafsson Liljenthal, Isabella Görloff Nordenskjöld, Martina Jarnlo Klarén, Andreas Ekman

**Affiliations:** ^1^ Department of Anaesthesia and Intensive Care Danderyd Hospital Stockholm Sweden; ^2^ Department of Clinical Sciences Danderyd Hospital, Karolinska Institutet Stockholm Sweden; ^3^ Collaboration for Evidence‐Based Practice and Research in Anaesthesia (CEPRA) Copenhagen Denmark; ^4^ Department of Paediatric and Obstetric Anaesthesia Aarhus University Hospital Aarhus Denmark; ^5^ Department of Anaesthesiology Nordsjællands Hospital Hillerød Denmark; ^6^ Department of Anaesthesiology Juliane Marie Centre Copenhagen Denmark; ^7^ Department of Clinical Medicine University of Copenhagen Copenhagen Denmark; ^8^ Department of Anaesthesiology Surgery and Trauma Centre Copenhagen Denmark; ^9^ Department of Anaesthesiology Sygehus Lillebælt Kolding Denmark; ^10^ Department of Regional Health Research University of Southern Denmark Odense Denmark

**Keywords:** anaesthesia, general intubation, intratracheal laryngoscopy, neuromuscular blocking agents, remifentanil

## Abstract

**Background:**

Neuromuscular blocking agents (NMBA) provide optimal conditions for tracheal intubation. A high dose of opioid can be used as an alternative but may give suboptimal conditions for intubation. The use of a video laryngoscope for intubation might eliminate this potential difference.

**Methods:**

A survey examining clinical practice regarding tracheal intubation with and without NMBA and use of video laryngoscopes was conducted in Sweden and Denmark.

**Results:**

1771 out of 3181 (56%) of invited anaesthetists responded. Overall, 1365/1771 (77%) preferred using NMBAs for non‐acute tracheal intubation with a considerably higher NMBA preference in Sweden 1011/1073 (94%) than in Denmark 354/700 (51%). A high proportion of Danish anaesthetists 327/700 (47%) compared to 40/1071 (4%) of Swedish anaesthetists reported primarily using opioids without NMBA. Remifentanil was the preferred opioid (1158/1361 (85%)) for intubation without NMBA. The reasons for using NMBA were improved intubating conditions (948/1771 (54%)), departmental tradition (285/1771 (16%)), adherence to local guidelines (264/1771 (15%)), and adherence to national/international guidelines (143/1771 (8%)). Video laryngoscopes were present in every operating theatre for 349/700 (50%) of Danish anaesthetists and 131/1071 (12%) of Swedish anaesthetists. Video laryngoscopes were easily accessible outside the operating room for 350/700 (50%) of Danish anaesthetists and 940/1071 (88%) of Swedish anaesthetists.

**Conclusions:**

NMBA use remains the standard for non‐acute tracheal intubation. However, a substantial number of anaesthetists regularly employ a NMBA‐free approach facilitated by high‐potency opioids and video laryngoscopy, particularly in Denmark. These findings emphasise the need for further research and subsequently updated evidence‐based guidance to support safe and effective intubation practices.

**Editorial Comment:**

This study, reporting a survey of anesthesia practitioners in Sweden and Denmark assessed preferences concerning how the respondents would manage a series of case scenarios concerning anesthetic drug choices for facilitation of intubation and employment of video laryngoscopy. Quite a bit of variation for preferences is presented by the responses concerning neuromuscular blockade or not to facilitate intubation.

## 1 Introduction

Airway management, including tracheal intubation, remains the leading cause of anaesthesia‐related morbidity and mortality [1, 2]. Globally, approximately one million patients are intubated each day [3]. Neuromuscular blocking agents (NMBAs) have traditionally been used to optimise conditions for intubation [4] and their use is recommended in international guidelines [5, 6, 7]. However, NMBAs are also associated with adverse effects, such as post‐anaesthesia respiratory complications [8], awareness [9], and anaphylaxis [10].

As an alternative, high‐potency opioids are used to facilitate tracheal intubation without the addition of NMBAs. Remifentanil, the most frequently used high‐potency opioid in this context, also has potentially serious side effects, including bradycardia and hypotension [11].

Notably, previous results from high‐quality trials comparing intubation with NMBA or remifentanil have been inconsistent. A recent systematic review suggested comparable intubation conditions between NMBAs and remifentanil [12]. However, these findings were based on a limited number of studies with various primary outcomes and definitions of difficult tracheal intubation, small sample sizes, and a moderate to high risk of bias. Previous studies, including meta‐analyses, have reported inferior intubation conditions when NMBAs were avoided during induction [4, 13, 14]. A recently published large, randomised trial comparing NMBA with remifentanil in rapid sequence induction showed a small but statistically significant difference, with remifentanil being inferior to NMBA regarding first pass success on tracheal intubation without major complications [15].

It is essential to assess how the expanding body of evidence affects clinical practice regarding tracheal intubation and how high‐potency opioids, without NMBAs, are used in clinical practice [16].

Video laryngoscopes have now become widely accessible and are commonly used as a first choice device or an alternative to traditional direct laryngoscopy [17, 18, 19, 20]. Video laryngoscopy greatly improves first‐pass intubation success [20, 21] and the use of video laryngoscopes as a first choice device is therefore strongly recommended in clinical guidelines [7, 22, 23, 24, 25]. Whether the improved view of the glottis obtained by video laryngoscopy eliminates the difference in first‐pass intubation success rate between using NMBAs or high‐potency opioids only is unknown. Also, it remains unclear whether the increased access to video laryngoscopes affects anaesthesia induction strategies in clinical practice.

The aim of this study was to describe current clinical practice on non‐acute tracheal intubation in anaesthesia departments in Denmark and Sweden. The primary objective was to determine the proportion of anaesthetists who primarily use NMBA or a high‐potency opioid to facilitate tracheal intubation. A secondary objective was to describe the availability and use of video laryngoscopes.

## 2 Methods

### 2.1 Study Design

We conducted a cross‐sectional web‐based survey in Denmark (DEN) and Sweden (SWE) to investigate current anaesthesia practices concerning the use of NMBAs, opioids, and video laryngoscopes in the surgical setting. The survey was conducted nationwide in both countries and directed to all anaesthetists working in public hospitals involved in orotracheal intubation during general anaesthesia for surgery, treatment, or investigative procedures on a regular basis.

A study protocol was published online prior to data collection [26]. The survey was designed and is reported in accordance with current survey reporting guidelines [27].

The survey was developed in Danish and underwent pilot testing and content validation by six Danish anaesthetists at the anaesthetic departments of Vejle and Kolding Hospitals. Subsequently, it was translated into English and adapted for use in Sweden. Cognitive interviews were conducted with two anaesthetists, and thereafter it was pilot tested by four Swedish anaesthetists at the Department of Anaesthesia and Intensive Care, Danderyd Hospital. The survey was revised with minor changes according to the cognitive interviews and pilot testing. The full survey questionnaires, in Danish and English, are presented in Appendices [Link], [Link].

The survey consisted of two parts: an individual‐level and a departmental‐level questionnaire.

The individual‐level questionnaire included three sections and a total of 22 questions: Section 1: (Questions 1–7) regarded respondent demographics, for example, level of education, years of experience, place of employment. Section 2: (Questions 8–10) investigated availability and use of video laryngoscopes. Section 3: (Questions 11–22) evaluated medication preferences for non‐acute tracheal intubation and included both general questions and three case‐based scenarios with closed‐end response options. These hypothetical scenarios covered anaesthesia preferences for three surgical cases: (a) elective arthroscopic knee surgery in a healthy young adult; (b) robot‐assisted colon resection in a healthy adult; (c) tonsillectomy in a 3‐year‐old child. All the above‐described questions were mandatory. Additionally, there were two optional questions with the possibility to add free text with comments before submitting the complete questionnaire.

The departmental level questionnaire contained seven questions and gathered information on institutional access to video laryngoscopes, annual volume of anaesthesia or surgical procedures (volume of anaesthesia cases for Denmark and surgical procedures for Sweden), staffing levels (i.e., number of employed anaesthetists), and the presence and content of local guidelines. Pre‐appointed local site investigators answered the departmental level questions.

### 2.2 Study Population

Eligible participants included anaesthetists employed at public hospitals in Denmark and Sweden who regularly administered general anaesthesia for surgery, treatment or investigative procedures. The survey was carried out using the secure Research Electronic Data Capture (REDCap, Vanderbilt, Nashville, TN, USA) [28] hosted by The Region of Southern Denmark.

### 2.3 Survey Distribution

A designated site investigator, responsible for identification of eligible colleagues and distribution of the survey, was appointed in each of the 37 Danish and 77 Swedish participating departments. Site investigators were informed that they were to be acknowledged as collaborators before agreeing to take part in the study. Each site investigator received unique distribution links for both the individual and departmental level questionnaires. Prior to distribution, site investigators were instructed to present a short 5‐min informational video to their colleagues introducing the study. Separate videos were produced for Denmark (by L.H.‐B.) and Sweden (by Å.L.) and made available online throughout the survey period at www.cepra.nu/ROCVIDEO. In Sweden, the survey was also advertised on the official website of the Swedish Association for Anaesthesia and Intensive Care [29]. Participation was anonymous for individual respondents; however, responses from site investigators were identifiable due to disclosure of workplace affiliation. The data collection period was from December 2024 through March 2025 in Denmark and from January to March 2025 in Sweden. Two reminder emails were sent: one after 2 weeks and another after approximately 6 weeks if the departmental response rate remained below 70%.

### 2.4 Data Collection and Management

Ethical approval was not required since patient data was not collected. Informed consent was obtained electronically; respondents confirmed their willingness to participate by selecting “yes” in response to the initial question of the survey. Data confidentiality was ensured through restricted access to the survey database, secured by username and a two‐step authentication with password and a personal identification number. Access was limited to three members of the research team (L.H.‐B., Å.L., and E.O.I.).

Only completed responses were included in the final analysis. This was defined as survey responses where the respondent filled out all questions and submitted the questionnaire as ‘complete’. Incomplete entries, identical duplicates, and responses from individuals not regularly providing anaesthesia for surgery, treatment or investigative procedures were excluded. Incomplete entries were recorded in the database if the survey was only partially filled out, despite most questions being mandatory (e.g., if an interruption occurred during questionnaire completion). Duplicate responses were possible to achieve since the survey was anonymous and access granted through a web link that did not require identification of the individual.

Free‐text fields were reviewed and categorised where relevant. Outliers in continuous variables were examined and excluded if deemed implausible.

### 2.5 Data Presentation and Statistical Analysis

The survey was designed with mandatory responses to nearly all questions. Two questions with free text fields were voluntary. Hospital of employment was not mandatory in order to increase respondent anonymity in regions with few anaesthetists.

There were no missing data except for 13 respondents not reporting the hospital where they were employed.

Descriptive statistics were used to summarise the data. Categorical variables are reported as counts and percentages. Continuous variables are presented as means with standard deviations for normally distributed data, and medians and interquartile ranges (IQR) or total range [MIN–MAX] for non‐normally distributed data. Country‐specific results are presented as subgroups. The Wilcoxon signed rank test was used to compare video laryngoscope use (ordinal options; 100%, 75%, 50%, 25%, 0%) when using NMBA or not.

As this was a convenience sample, no formal sample size calculation was performed. All statistical analyses were performed using R and RStudio. Choropleth maps were created using Microsoft Excel.

## 3 Results

### 3.1 Respondent Characteristics

**FIGURE 1:**
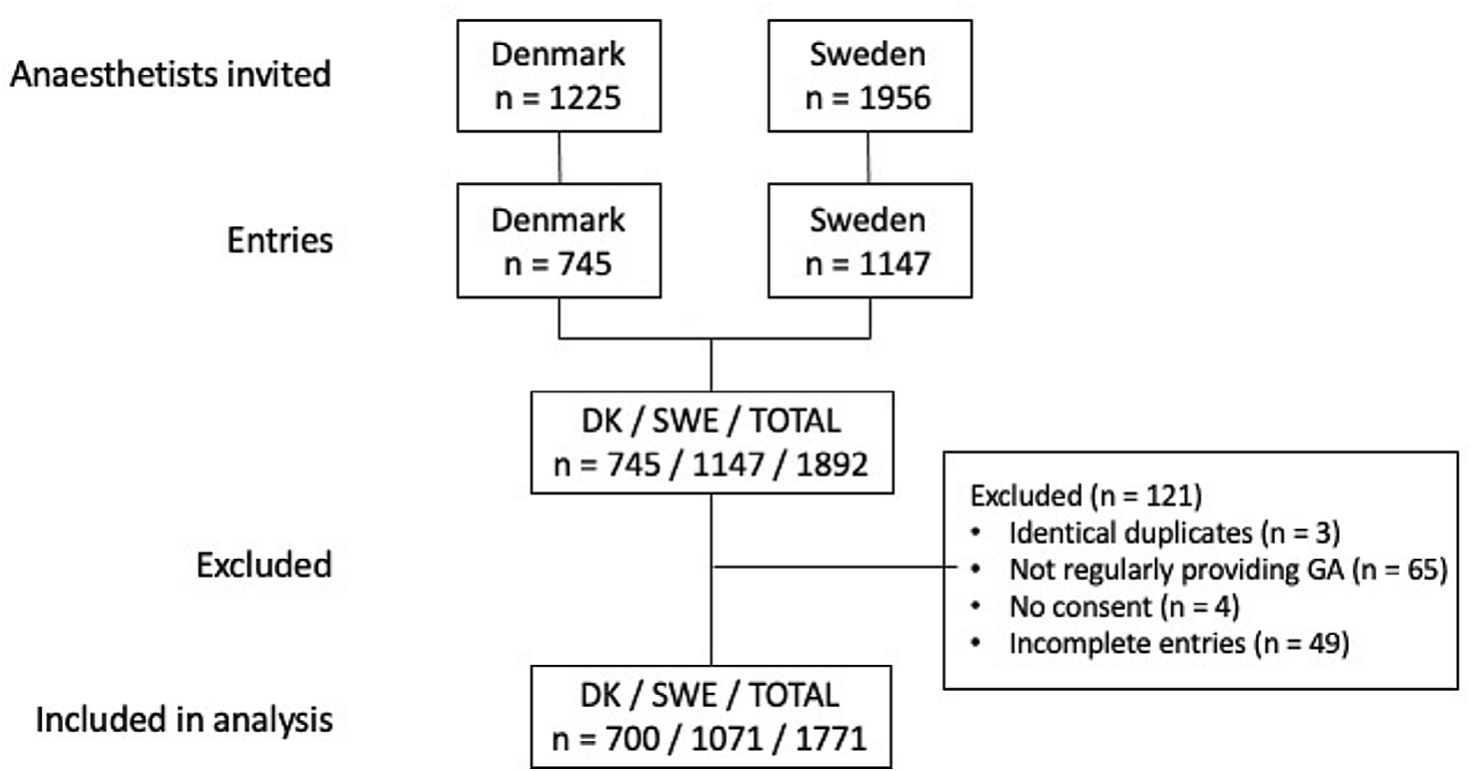
Participant flow chart of the survey.

Valid responses were received from 1771 participants out of 3181 invited, resulting in a response rate of 56% (Figure 1).

Respondent characteristics are summarised in Table 1. Details regarding the region of employment of respondents and anaesthesia for the various types of surgery are presented in Appendices S5 and S6.

**TABLE 1:** Characteristics of the survey respondents.

	Denmark (*N* = 700)	Sweden (*N* = 1071)	Overall (*N* = 1771)
Age			
Median [Min, Max]	45 [28–73]	43 [27–78]	44 [27–78]
Sex			
Male	417 (60%)	609 (57%)	1026 (58%)
Female	279 (40%)	456 (43%)	735 (42%)
Would prefer not to specify	0 (0%)	6 (1%)	6 (0%)
Other	4 (1%)	0 (0%)	4 (0%)
Education level			
Pre‐registration house officer	1 (0%)	1 (0%)	2 (0%)
Fully registered medical practitioner	0 (0%)	4 (0%)	4 (0%)
Registrar	178 (25%)	268 (25%)	446 (25%)
Specialist	521 (74%)	798 (75%)	1319 (74%)
Years of experience			
< 2 years	67 (10%)	74 (7%)	141 (8%)
2–5 years	107 (15%)	204 (19%)	311 (18%)
6–10 years	114 (16%)	205 (19%)	319 (18%)
11–15 years	136 (19%)	178 (17%)	314 (18%)
16–20 years	110 (16%)	135 (13%)	245 (14%)
20 years	166 (24%)	275 (26%)	1000 (25%)

Respondents represented all geographical regions of Denmark and Sweden, except for the Kronoberg Region in Sweden, who declined participation. Departments of all sizes were included measured by number of consultants: (20 [3–85]), number of operating theatres (12 [4–54]), and annual anaesthesia or surgical case volumes (11,000 [1000–40,000] and 6100 [1400–29,000], for Denmark and Sweden, respectively).

The Stockholm‐Gotland region in Sweden had the highest overall number of respondents (266/1771, 15%) followed by Mellan‐Sverige (234/1771, 13%) and The Capital region of Denmark (229/1771, 13%). The North Denmark Region was least represented (69/1771, 4%).

### 3.2 Pharmacological Strategies for Non‐Acute Tracheal Intubation

**FIGURE 2:**
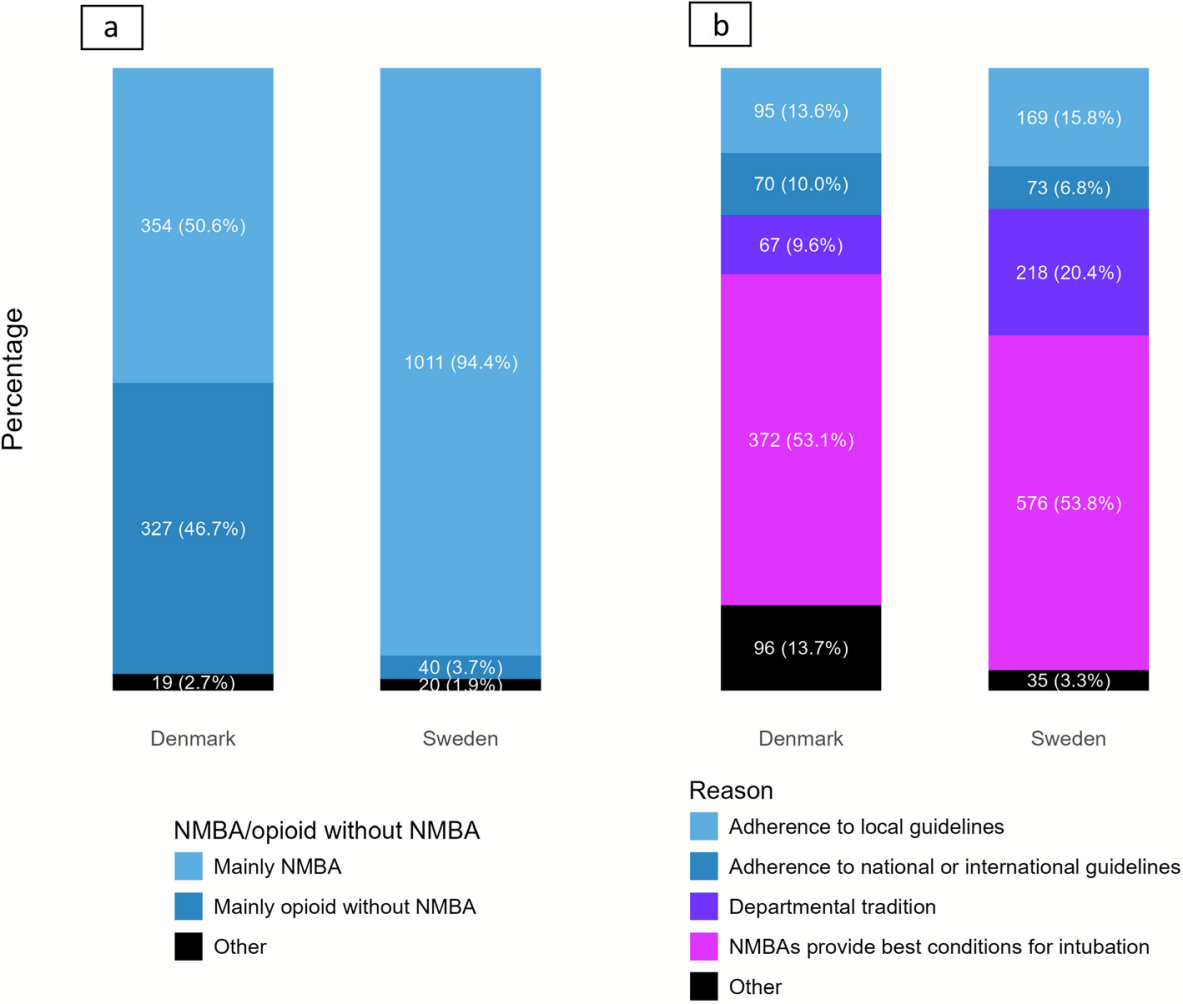
(a) Routine for pharmacological strategy in non‐acute orotracheal intubation in Denmark and Sweden with use of neuromuscular blocking agent (NMBA) versus opioids without NMBA. (b) The reasons for choosing NMBA among all participants, including those who primarily chose to intubate without NMBA. Comments to “Other”: “depending on whether the surgery requires neuromuscular block”, “depending on whether the airway seems difficult to manage”, “I use NMBA vs opioids in 50%/50%”,” if the vocal cords are insufficiently open without NMBA”, “If the patient can't handle a high‐dose of opioid (e.g., heart disease)” or “For educational purposes”.

**FIGURE 3:**
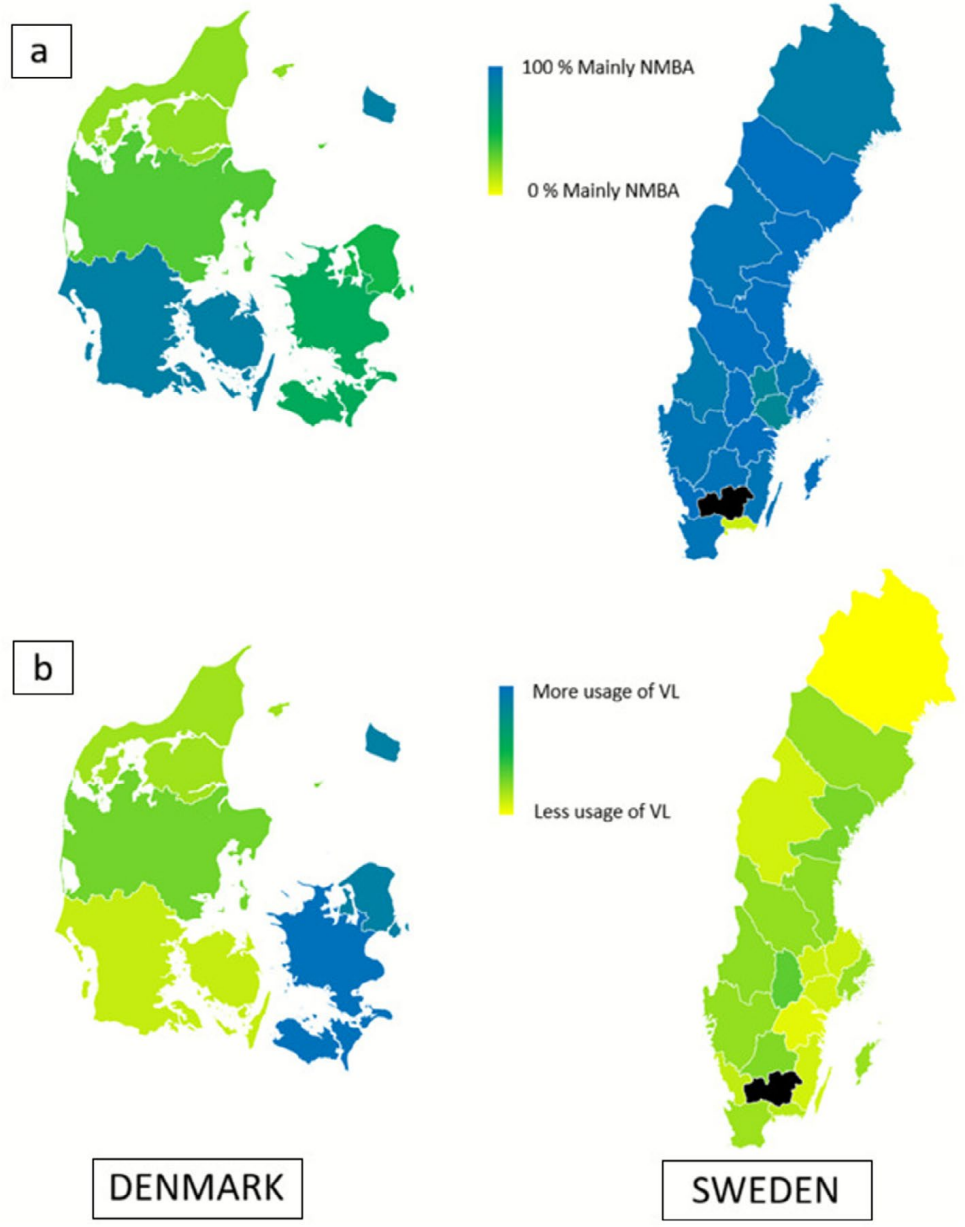
Choropleth map showing regional differences in Denmark and Sweden regarding: (a) use of neuromuscular blocking agents (NMBAs) (options “I mainly use NMBAs” or “I most often use opioid without NMBA”). Here displayed as the percentage of respondents answering “I mainly use NMBAs”. (b) Use of video laryngoscope. Answering the question “Which option best describes to what extent you use a video laryngoscope for non‐acute intubations?” Data displayed as weighted means of ordinal options (100%, 75%, 50%, 25% and 0%). Region with black marking (Kronoberg) did not participate in the study.

Overall, (1365/1771 (77%)) of respondents reported routinely using NMBAs for non‐acute tracheal intubation. The proportion of NMBA use was considerably higher in Sweden (1011/1071 (94%)) than in Denmark (354/700 (51%)) (Figure 2a), with considerable regional variability within Denmark (Figure 3a). There was no pronounced difference in NMBA use when comparing anaesthetists with varying degrees of training, < 2 years, 2–5 years, 6–10 years, 11–15 years, 16–20 years, or ≥ 20 years of practice (Table 2).

**TABLE 2:** Use of neuromuscular blocking agents (NMBA) versus opioid without NMBA as a routine pharmacologic strategy for tracheal intubation in relation to years of anaesthesia practice of respondents.

	< 2 years	2–5 years	6–10 years	11–15 years	16–20 years	> 20 years	Overall
Mainly NMBA	107 (76%)	248 (80%)	262 (82%)	238 (76%)	178 (73%)	332 (75%)	1365 (77%)
Mainly opioid without NMBA	29 (21%)	55 (18%)	54 (17%)	69 (22%)	66 (27%)	94 (21%)	367 (21%)
Other	5 (4%)	8 (3%)	3 (1%)	7 (2%)	1 (0%)	15 (3%)	39 (2%)

*Note:* Data presented as number (proportion).

All participants stated reasons for using NMBA regardless of their routine pharmacological strategy, including the 327 participants who preferred to use only opioids as a routine for tracheal intubation. The reasons for using NMBA were improved intubating conditions (948/1771 (54%)), departmental tradition (285/1771 (16%)), adherence to local guidelines (264/1771 (15%)), and adherence to national/international guidelines (143/1771 (8%)). (Figure 2b).

When using NMBA, rocuronium was the most frequently chosen NMBA for routine intubation, reported by 1630/1771 (92%) of all respondents. Suxamethonium was reported as the second‐most used NMBA by 119/1771 (7%) (Table 3).

**TABLE 3:** Choice of neuromuscular blocking agent (NMBA) and opioid without NMBA for non‐acute orotracheal intubation.

	Denmark (*N* = 700)	Sweden (*N* = 1071)	Overall (*N* = 1771)
Preferred NMBA for non‐acute intubations			
Rocuronium	605 (86%)	1025 (96%)	1630 (92%)
Suxamethonium	79 (11%)	40 (4%)	119 (7%)
Cisatracurium	10 (1%)	3 (0%)	13 (1%)
Other	6 (1%)	3 (0%)	9 (1%)
Preferred opioid (without concomittant NMBA) for non‐acute intubations			
Remifentanil	573 (82%)	585 (55%)	1158 (65%)
Fentanyl	52 (7%)	68 (6%)	120 (7%)
Alfentanil	7 (1%)	58 (5%)	65 (4%)
Sufentanil	6 (1%)	4 (0%)	10 (1%)
I never/almost never use an opioid without NMBA for intubation	61 (9%)	351 (33%)	412 (23%)
Other	1 (0%)	5 (0%)	6 (0%)

*Note:* Data presented as number (proportion).

Remifentanil was the preferred opioid (1158/1771 (65%)) for most of the respondents when choosing to intubate with an opioid without using NMBA (Table 3).

### 3.3 Use of and Availability of Video Laryngoscopes

**FIGURE 4:**
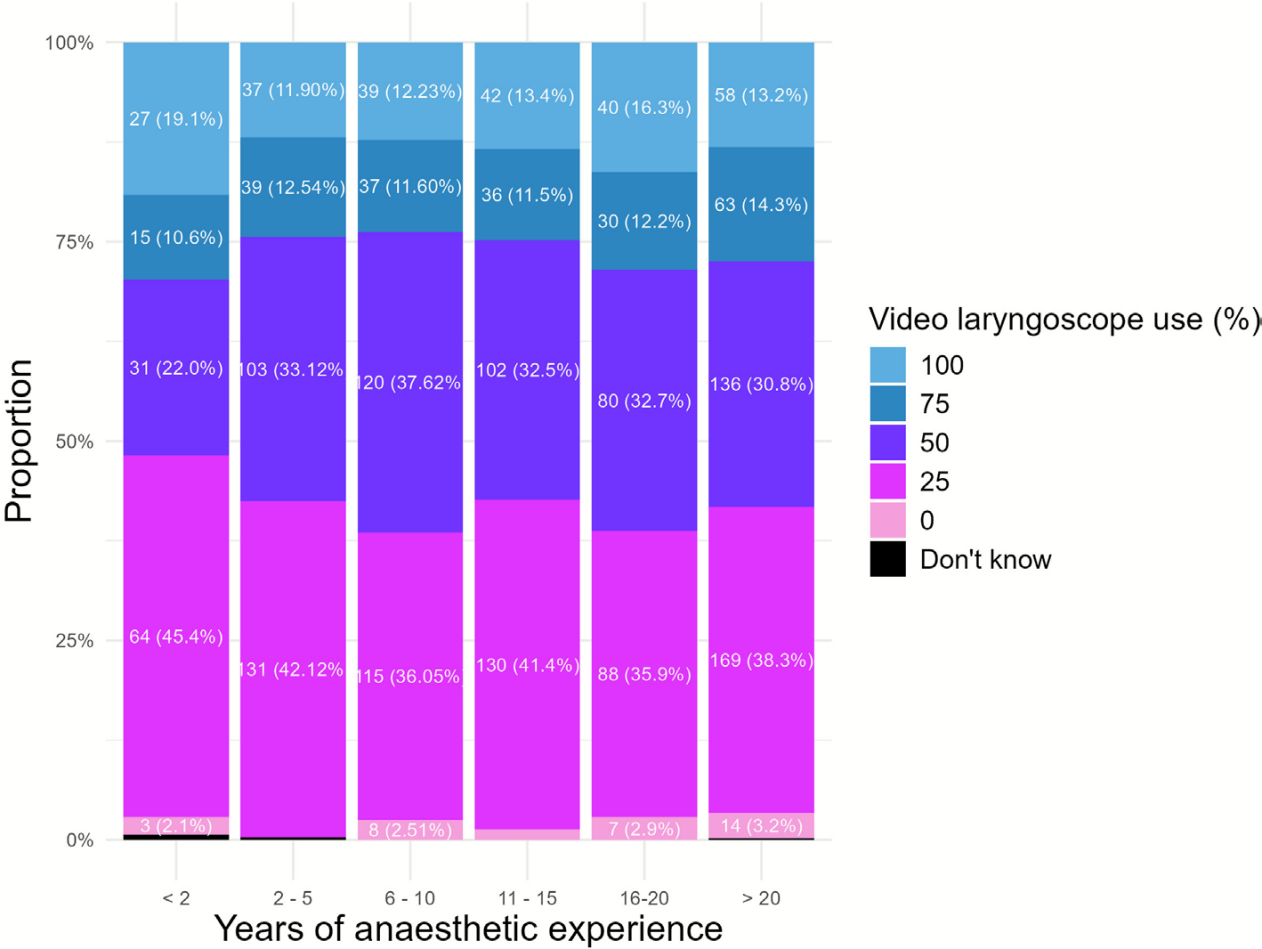
Bar chart showing the proportion of respondents using the video laryngoscope in 0%, 25%, 50%, 75% and 100% of tracheal intubations in relation to years of anaesthesia working experience. One respondent in categories < 2 years (0.7%), 2–5 years (0.3%) and > 20 years (0.2%) of working experience stated “Don't know” to what extent they used a video laryngoscope.

Reported use of video laryngoscopes varied within and between the two countries (Figure 3b). Video laryngoscopes were present in every operation theatre for 50% of Danish anaesthetists as opposed to 12% in Sweden. Video laryngoscopes were easily accessible outside the operating theatre for 350/700 (50%) of Danish and 940/1071 (88%) of Swedish anaesthetists thus making video laryngoscopes easily accessible for 100% of anaesthetists in both countries, but present in every operation theatre in a considerably higher degree in Denmark than in Sweden. The proportion of anaesthetists using the video laryngoscope in 25%, 50%, 75% or 100% of cases did not particularly differ between groups with varying time of anaesthetic training (Figure 4). Reasons for using the video laryngoscope were: when indicated 1320/1771 (75%), “I prefer the video laryngoscope” 251/1771 (14%), for educational purposes 67/1771 (4%), “the only available laryngoscope” 34/1771 (2%). 99/1771 (6%) of respondents stated “other reason.” Comments to “other reason” were “I consider video laryngoscopy safer for the patient,” “I use video laryngoscope whenever it is available,” “I use the video laryngoscope for multiple reasons,” “I adhere to the departmental guidelines,” “I primarily use the video laryngoscope but sometimes use direct laryngoscopy to maintain the skill.”

The proportion of reported video laryngoscope use differed significantly between respondents who primarily used NMBAs for tracheal intubation and those who did not (*p* < 0.001). Respondents who frequently used video laryngoscopes reported less frequent use of NMBAs. This difference was also seen in the subgroup analysis for each country (Tables 4 and 5) (Denmark, *p* = 0.002; Sweden, *p* = 0.049).

**TABLE 4:** Usage of video laryngoscope (VL) versus pharmacological routine for non‐acute tracheal intubation in Denmark.

VL usage	Mainly NMBA (*n* = 354)	Mainly opioid without NMBA (*n* = 327)	Other (*n* = 19)	Overall (*n* = 700)
100%	106 (30%)	105 (32%)	8 (42%)	219 (31%)
75%	33 (9%)	57 (17%)	2 (11%)	92 (13%)
50%	95 (27%)	102 (31%)	4 (21%)	201 (29%)
25%	117 (33%)	61 (19%)	4 (21%)	182 (26%)
0%	3 (1%)	2 (1%)	2 (1%)	5 (1%)
Don't know	0 (0%)	0 (0%)	1 (5%)	1 (0%)

**TABLE 5:** Usage of video laryngoscope (VL) versus pharmacological routine for non‐acute tracheal intubation in Sweden.

VL usage	Mainly NMBA (*n* = 1011)	Mainly opioid without NMBA (*n* = 40)	Other (*n* = 20)	Overall (*n* = 1071)
100%	22 (2%)	0 (0%)	2 (10%)	24 (2%)
75%	127 (13%)	1 (2%)	0 (0%)	128 (12%)
50%	350 (35%)	14 (35%)	7 (35%)	371 (35%)
25%	481 (48%)	23 (58%)	11 (55%)	515 (48%)
0%	30 (3%)	1 (2%)	0 (0%)	31 (3%)
Don't know	1 (0%)	1 (2%)	0 (0%)	2 (0%)

### 3.4 Case‐Scenario Responses

Summary results for all three case scenarios are presented in Table 6. In an elective knee surgery scenario, 379/1773 (21%) of respondents reported that they would avoid using NMBA. In a laparoscopy case, 68/1773 (4%) would not use NMBA to facilitate intubation. In the paediatric tonsillectomy scenario, approximately one quarter (461/1773 (26%)) reported they would not use NMBAs.

**TABLE 6:** Summary of choice of neuromuscular blocking agent (NMBA), opioid and dosage of these drugs in three case‐based scenarios.

	Cases		
	Healthy young adult (70 kg)		Child (3 years, 15 kg)
	Arthroscopy	Laparoscopy	Tonsillectomy
Preferred NMBA for tracheal intubation^a^			
Rocuronium	1003 (57%)	1673 (94%)	859 (48%)
Suxamethonium	338 (19%)	18 (1%)	321 (18%)
Wouldn't use NMBA	385 (22%)	71 (4%)	487 (27%)
Other NMBA	25 (1%)	9 (0.5%)	104 (6%)
Dosage of rocuronium^b^	40 (38–42)	45 (40–50)	10 (9.0–10)
Preferred opioid without concomitant NMBA^c^			
Remifentanil	1170 (66%)	809 (46%)	737 (42%)
Fentanyl	140 (8%)	183 (10%)	294 (17%)
Alfentanil	61 (3%)	39 (2%)	133 (7%)
Wouldn't use without NMBA	376 (21%)	718 (41%)	560 (32%)
Other	24 (2%)	22 (2%)	47 (3%)
Dosage of remifentanil^d^	200 (150–280)	200 (140–280)	50 (30–60)

*Note:* Values presented as numbers (proportion), for dosage of rocuronium (median (IQR) mg) and for dosage of remifentanil (median (IQR) μg).

^a^ What NMBA would you give as bolus dose to facilitate tracheal intubation for elective knee arthroscopy?

^b^ For respondents answering “rocuronium”.

^c^ Which opioid would you give as a bolus dose to facilitate tracheal intubation, if you planned NOT to use a NMBA?

^d^ For respondents answering “remifentanil”.

Rocuronium was the most widely used NMBA for non‐acute tracheal intubation, reported by 859/1771 (49%). When intubating without NMBA remifentanil was the most frequently preferred drug, used by 737/1771 (42%) respondents.

Reported rocuronium dosages were relatively uniform with a median of 0.57 mg/kg (IQR: 0.54–0.6) for the arthroscopic case and 0.64 mg/kg (IQR: 0.6–0.71) for the laparoscopic case. In comparison, remifentanil dosing showed greater variability with a median of 2.9 μg/kg (IQR: 2.1–4.7) for the arthroscopic case and 2.9 μg/kg (IQR: 2.0–4.0) for the laparoscopic case.

## 4 Discussion

This large cross‐sectional survey of clinical practices among anaesthetists in Denmark and Sweden provides contemporary insight into clinical anaesthesia routine in Scandinavia, specifically regarding pharmacological strategies and use of video laryngoscopy for non‐acute tracheal intubation. Most respondents (77%) reported primarily using NMBAs, which aligns with current international guidelines for both the adult and paediatric population [5, 6, 7, 23].

However, a substantial proportion of anaesthetists, especially in Denmark, reported routine use of high‐potency opioids, most often remifentanil, without NMBAs to facilitate tracheal intubation. In fact, 47% of Danish respondents indicated that they primarily intubate without NMBAs, compared to only 4% in Sweden. This striking difference between countries suggests not only a divergence from established guidelines but also possibly reveals a shift in clinical practice in parts of the Danish anaesthesia community. These findings are consistent with previous reports [4, 13], suggesting a growing interest in NMBA‐free intubation, potentially driven by the risk of adverse events associated with NMBA use [12] and the convenience of NMBA‐free intubation. In contrast, Swedish practice appears more closely aligned with international and national guidelines [6, 30].

No previous combined data from Denmark and Sweden exist on the clinical use of NMBAs. However, data from 822,259 tracheal intubations performed in Denmark between 2008 and 2016, extracted from the Danish Anaesthesia Database, reveal that 71.1% of intubations in this period were facilitated by a NMBA [20].

Regional differences in the use of NMBA were seen, particularly in Denmark. There are three Danish Regions of specialty training, but the specialty training programme is centralised, giving every trainee the same formal education. Although bed‐side clinical training may vary according to local guidelines and preferences.

In Sweden one region, representing a relatively small number of anaesthetists (Blekinge), deviated from the prevailing practice of routinely using NMBAs for tracheal intubation. A possible explanation for this may be the influence of Danish anaesthetists who regularly work in the region on a temporary basis.

Interestingly, despite common use of NMBA‐free intubation, most respondents in both countries considered NMBAs to provide the best conditions for intubation. The high frequency of opioid‐only intubation in Denmark may reflect local tradition, lack of national guidelines or institutional cultures. This warrants further investigation.

The use and availability of video laryngoscopes varied markedly between countries (Figure 3b). Danish respondents reported a markedly higher availability and frequency of usage of video laryngoscopes compared with their Swedish colleagues. Given the similarities in healthcare systems and resources, these differences may reflect cultural norms, institutional policies, or variations in guideline emphasis rather than infrastructure or funding limitations. The 2024 Swedish airway management guidelines explicitly recommend the use of video laryngoscope for all tracheal intubations with rapid sequence induction; for caesarean sections in general anaesthesia; during education; and when intubating obese or critically ill patients. The guideline also recommends the use of a video laryngoscope as a first‐choice device whenever it is available [31].

One novel aspect of this study is the exploration of how video laryngoscopy use relates to pharmacological intubation strategies. The finding that anaesthetists who primarily perform tracheal intubation using a video laryngoscope reported considerably less frequent use of NMBA suggests that the video laryngoscope may be perceived as a compensatory tool for not achieving the intubation conditions provided by NMBA. This interpretation is supported by the finding that the most frequently reported reason for NMBA use was to optimise intubation conditions. Whether this practice is a product of individual or departmental choice is unknown.

The association between not using NMBA when utilising video laryngoscopy was observed in both subgroups, although the routine strategy in the two countries clearly differed, with a more widespread use of NMBAs among Swedish respondents.

### 4.1 Strengths and Limitations

Our study has several strengths, including a large, representative sample of anaesthetists from Denmark and Sweden, with wide geographical coverage and participation from departments of varied sizes, case mix and volumes (Appendix S6). To our knowledge, this is the first study to systematically examine large‐scale real‐world use of NMBAs, opioids and video laryngoscopes for tracheal intubation across an entire anaesthesia workforce as previously recommended [16].

However, the study also has limitations. First, the survey was designed to be easily distributed and accessible, not requiring individual identification of the participants to maximise response rate and anonymity. This resulted in a small number of duplicates that were found during analysis. Undetected duplicates cannot be ruled out. Second, while we focused on preferred and routine practices, we could not validate actual behaviour in the clinical setting. Third, while we asked for the reason to use NMBA, we did not inquire about the reason to avoid it, using opioids only for intubation. We assumed that the reasons for avoiding NMBA were for convenience and to avoid potential adverse effects. However, other factors, such as a high cost of neuromuscular block reversal with sugammadex, cannot be ruled out. Finally, the cross‐sectional nature of the survey precludes conclusions about causality or temporal trends.

We noted potential ambiguity in the responses to the case scenario involving knee arthroscopy in a healthy adult. Some respondents may have interpreted this scenario as one suitable for a supraglottic device rather than tracheal intubation, which may have influenced their reported pharmacological approach. Several participants commented on this ambiguity.

### 4.2 Clinical Implications and Future Directions

Our findings highlight a need for evidence‐based data on opioid‐based intubation techniques and guidance on when NMBA‐free strategies may be appropriate. Such evidence could support the development of differentiated guidelines tailored to specific patient populations, procedures, and institutional practices. The evolving role of the video laryngoscope—especially as a potential enabler of NMBA‐free intubation—should be more thoroughly explored in clinical trials.

## 5 Conclusion

While NMBAs remain the standard for non‐acute tracheal intubation, our findings reveal a substantial number of anaesthetists, particularly in Denmark, who regularly employ a NMBA‐free approach. This is instead facilitated by high‐potency opioids and, to a certain degree, video laryngoscopy. These findings emphasise the need for further research followed by updated evidence‐based guidance to support safe, effective, and individualised intubation practices.

## Author Contributions

Åse Lodenius and Louise Holland‐Bill both contributed equally. Study design: L.H.‐B., H.K.N., A.K.N. Translation: Å.L. Survey distribution and data collection: L.H.‐B., Å.L. Analysing data: Å.L., L.H.‐B., E.Ø.I., H.K.N., A.K.N. Data visualisation: E.Ø.I. Drafting the article: E.Ø.I., Å.L. Revising the article: Å.L., L.H.‐B., E.Ø.I., M.C., A.A., M.V., A.C., L.H.L., H.K.N., A.K.N. Final approval of the article: all authors.

## Funding

The authors have nothing to report.

## Conflicts of Interest

The authors declare no conflicts of interest.

## Supporting information


**APPENDIX S1:** Danish questionnaire for individuals.

**APPENDIX S2:** Danish questionnaire for departments.

**APPENDIX S3:** English questionnaire for individuals.

**APPENDIX S4:** English questionnaire for departments.

**APPENDIX S5:** Region of employment.

**APPENDIX S6:** Types of surgery.

## Data Availability

The data that support the findings of this study are available from the corresponding author upon reasonable request.
