# Supplementary material for: Neuromuscular Block and Video Laryngoscope to Facilitate Intubation—A Survey of Current Practice in Denmark and Sweden
Source: Acta Anaesthesiol Scand. 2026 Mar 13;70(4):e70200. doi: 10.1111/aas.70200 (PMC12983051; doi:10.1111/aas.70200)
Supplement: Supplementary file 1 — APPENDIX S1: Danish questionnaire for individuals. [file AAS-70-0-s004.pdf]

Individ niveau

Venligst udfyld nedenstående spørgeskema

Tak!

Det følgende spørgeskema indgår i det skandinaviske forskningsprojekt ROCVIDEO*, og søger kort fortalt at afdække den aktuelle tilgang til ikke-akutte intubationer på operationsgangene på danske offentlige hospitaler, herunder især valg af induktionsmedicin og brugen af videolaryngoskoper til facillitering af ikke-akut intubation, og determinanter med betydning for disse valg.

Den fulde protokol er offentlig tilgængelig via Open Science Framework (https://osf.io/rv7jg) eller kan tilgås på nedenstående link.

Forskningsprojektet udgår fra CEPRA**

Spørgeskemaet tager ca.10 minutter at færdiggøre, og din besvarelse er anonym.
Vi håber, at du har lyst til at hjælpe med at afdække den nuværende kliniske praksis, og takker for din tid.

Mange hilsner
ROC-VIDEO forskningsgruppen

*ROCVIDEO er et selvstændigt forskningssamarbejde søger at undersøge fordele og risici ved at anvende hurtigtvirkende, høj-potent opioid (remifentanil) versus muskel relaksans (rocuronium) til optimering af intubations-forholdene hos patienter som undergår videolaryngoskopi-assisteret trakeal intubation.

**CEPRA er et nationalt perioperativt forskningsnetværk bredt repræsenteret af forskere og afdelinger i Danmark. Formålet er at fremme nationalt forskningssamarbejde af høj kvalitet. (www.cepra.nu).

[Attachment: "Protocol - Danish national survey on current practice Non-RSI intubation.pdf"]

Er du Site-investigator på din afdeling?

◯ Nej
◯ Ja

SAMTYKKEERKLÆRING

Jeg har læst ovenstående beskrivelse af spørgeskemaet.

Jeg er indforstået med, at mine besvarelser er fuldt anonymiserede.

Jeg er også indforstået med, at min samtykke til enhver tid kan tilbagekaldes, hvilket gøres ved at afslutte spørgeskemaet/forlade hjemmesiden uden at indsende svarene

◯ Ja, jeg samtykker hermed til at deltage i studiet
◯ Nej, jeg samtykker ikke til at deltage i studiet

DEL 1 - DEMOGRAFI

Dit primære ansættelsessted er	◯ på offentligt sygehus<br>◯ på privat sygehus<br>◯ jeg arbejder lige meget på offentligt og privat sygehus<br>◯ præhospitalt<br>◯ Andet
Uddyb venligst	__________________________________
Arbejder du udelukkende præhospitalt?	◯ Ja<br>◯ Nej
Vælg venligst det udsagn som passer bedst på dit anæstesiologiske arbejde	◯ Jeg er tilknyttet Intensiv og varetager kun sjældent anæstesi til operation, behandling eller undersøgelse.<br>◯ Størstedelen af mit arbejde er at varetage anæstesi til operation, behandling eller undersøgelse.<br>◯ Jeg varetager både intensiv terapi og anæstesi til operation, behandling eller undersøgelse.<br>◯ Ved ikke
Hvilket/hvilke speciale(r) bedøver du hyppigt til? (flere valg er mulige)	☐ Børn og unge<br>☐ Obstetrik kirurgi<br>☐ Gynækologisk kirurgi<br>☐ Mavetarm-kirurgi<br>☐ Urologisk kirurgi<br>☐ Ortopædkirurgi<br>☐ Thoraxkirurgi<br>☐ Neurokirurgisk<br>☐ Øre-næse-hals-kirurgi<br>☐ Plastikkirurgi<br>☐ Dagkirurgi<br>☐ Andet
Uddyb venligst	__________________________________
Angiv venligst dit uddannelsesniveau	◯ Uklassiciceret uden forudgående Introuddannelse<br>◯ Introuddannelse<br>◯ Uklassificeret efter introuddannelse<br>◯ Hoveduddannelse<br>◯ Speciallæge
Hvor mange år har du arbejdet indenfor anæstesien?	◯ < 2 år<br>◯ 2-5 år<br>◯ 6-10 år<br>◯ 11-15 år<br>◯ 16-20 år<br>◯ > 20 år
Angiv venligst dit køn	◯ Mand<br>◯ Kvinde<br>◯ Andet
Angiv venligst din alder (i år)	__________________________________

I hvilket land er dit primære ansættelsessted?	◯ Færøerne<br>◯ Grønland<br>◯ Danmark
I hvilken region i Grønland er du primært ansat?	◯ Ilulissat<br>◯ Aasiaat<br>◯ Sisimiut<br>◯ Nuuk<br>◯ Qaqortoq<br>◯ Andet
Uddyb venligst	______________________________
På hvilket sygehus på Færøerne arbejder du primært?	◯ Landssjúkrahúsið<br>◯ Klaksvík Hospital<br>◯ Suðuroyar Sjúkrahús<br>◯ Andet
Uddyb venligst	______________________________
I hvilken region er du ansat (primære ansættelsessted)?	◯ Region Nordjylland<br>◯ Region Midtjylland<br>◯ Region Syddanmark<br>◯ Region Hovedstaden<br>◯ Region Sjælland
På hvilket offentligt sygehus i Region Nordjylland er du ansat?	◯ Aalborg Universitets hospital, Syd<br>◯ Aalborg Universitets hospital, Nord<br>◯ Farsø Hospital<br>◯ Hobro Hospital<br>◯ Thisted Sygehus<br>◯ Frederikshavn/Hjørring (Regionshospital Nordjylland)
På hvilket offentligt sygehus i Region Midtjylland er du ansat?	◯ Aarhus Universitetshospital.<br>◯ Regionshospitalet Gødstrup.<br>◯ Regionshospitalet Horsens.<br>◯ Regionshospitalet Randers.<br>◯ Regionshospitalet Silkeborg.<br>◯ Regionshospitalet Viborg.<br>◯ Regionshospitalet Skive og Hammel
På hvilket offentligt sygehus i Region Syddanmark er du ansat?	◯ Esbjerg Sygehus.<br>◯ Odense Universitetshospital.<br>◯ Svendborg Sygehus.<br>◯ Sygehus Lillebælt, Kolding.<br>◯ Sygehus Lillebælt, Vejle.<br>◯ Sygehus Sønderjylland, Sønderborg.<br>◯ Sygehus Sønderjylland, Åbenrå.

På hvilket offentligt sygehus i Region Hovedstaden er du ansat?	◯ Amager Hospital<br>◯ Bispebjerg Hospital<br>◯ Bornholms Hospital<br>◯ Frederiksberg Hospital<br>◯ Gentofte Hospital<br>◯ Herlev Hospital<br>◯ Hvidovre Hospital<br>◯ Nordsjællands Hospital<br>◯ Frederikssund Sygehus<br>◯ Rigshospitalet - Blegdamsvej<br>◯ Rigshospitalet - Glostrup
På hvilket offentligt sygehus i Region Sjælland er du ansat?	◯ Sjællands Universitetshospital, Køge<br>◯ Sjællands Universitetshospital, Roskilde<br>◯ Holbæk Sygehus<br>◯ Sjællands Universitetshospital, Nykøbing F<br>◯ Næstved Sygehus<br>◯ Slagelse Sygehus<br>◯ Ringsted Sygehuse

DEL 2 - VIDEOLARYNGOSKOPI

Vi vil nu gerne høre lidt om din brug af videolaryngoskop til ikke-akutte intubationer.

Er der på din afdeling adgang til videolaryngoskoper?	◯ Ja, de ligger på alle operationsstuer.<br>◯ Ja, jeg har let adgang til et videolaryngoskop, men de ligger ikke på alle operationsstuer.<br>◯ Nej, jeg ikke let adgang til videolaryngoskop.<br>◯ Nej, jeg har overhovedet ikke adgang til videolaryngoskop.<br>◯ andet
Uddyb venligst	__________________________________
Hvilken valgmulighed beskriver BEDST i hvilket omfang du bruger videolaryngoskop til ikke-akutte intubationer?	◯ Jeg anvender stort set altid videolaryngoskop (ca. 100%)<br>◯ jeg anvender hyppigt videolaryngoskop (ca. 75%)<br>◯ jeg anvender videolaryngoskop og konventionel laryngoskop stort set lige meget (ca. 50%)<br>◯ jeg anvender kun sjældent videolaryngoskop (ca. 25%)<br>◯ Jeg anvender stort set aldrig videolaryngoskop (ca.0 %)<br>◯ ved ikke
Hvilken svarmulighed beskriver BEDST hvorfor du vælger videolaryngoskopi til ikke-akutte intubationer?	◯ Jeg anvender videolaryngoskop på indikation (hvis der er vurderet grad af vanskelig luftvej, nakkeproblemer, svær overvægt osv).<br>◯ Jeg anvender videolaryngoskop i uddannelses øjemed.<br>◯ Jeg anvender videolaryngoskop fordi det er det eneste tilgængelige laryngoskop<br>◯ Jeg foretrækker altid at bruge videolaryngoskop<br>◯ andet
Uddyb venligst	__________________________________

DEL 3 - INTUBATIONSMEDICIN

I denne del vil vi gerne vide lidt om dine preferencer i forhold til intubationsmedicin.

Bemærk at alle spørgsmål omhandler medicin til ikke-akutte intubationer.

Vigtigt: en bolus opioid kan enten gives som "single shot", ved kortvarigt at lade infusion på pumpen køre med en høj rate eller en kombination af de to.

Hvor ofte anvender du bolus neuromuskelært blokerende agenter (NMBA) versus bolus opioid (single shot, infusion med høj rate eller kombination) uden NMBA som facilitator ved ikke-akutte intubationer,?	◯ Jeg anvender overvejende NMBA<br>◯ Jeg anvender overvejende opioid uden NMBA.<br>◯ Andet
Beskriv venligst kort den anvendte metode	__________________________________
Hvilken type NMBA ville du typisk anvende til ikke-akut intubation?	◯ Suxamethon<br>◯ Rocuronium<br>◯ Cisatracurium<br>◯ Andet<br>(angiv det du oftest vælger)
Angiv venligst hvilket ..	__________________________________
Hvilket opioid ville du typisk anvende som bolus til ikke-akut intubation?	◯ Remifentanil<br>◯ Alfentanil<br>◯ Fentanyl<br>◯ Sufentanil<br>◯ Jeg anvender aldrig/næsten aldrig bolus opioid uden NMBA til intubation<br>◯ Andet<br>(angiv det du oftest vil vælge)
Angiv venligst hvilket præparat der er tale om...	__________________________________
For hvor stor en andel af ikke-akutte intubationer, hvor du anvender bolus opioid som primær facilitator for intubation, oplever du at skulle supplere med relaksans?	◯ 0-24%<br>◯ 25-49%<br>◯ 50-74%<br>◯ 75-100 %
Angiv hvilket udsagn der passer bedst: "jeg giver muskelrelaksans fordi....	◯ jeg følger afdelingens instruks<br>◯ jeg følger nationale/europæiske/internationale guidelines<br>◯ det er der tradition for i afdelingen<br>◯ det giver de bedste intubationsforhold.<br>◯ andet
Beskriv venligst hvorfor	__________________________________

Hvilket NMBA ville du anvende som bolus til anæstesi-induktion til en rask ung voksen på 70 kg, 170 cm høj, som skal have foretaget knæartroskopi (eller anden operation som ikke kræver peroperativ eller kirurg-ordineret relaksation)?"	◯ Suxamethonium<br>◯ Rocuronium<br>◯ Cisatracurium<br>◯ Mivacurium<br>◯ Jeg ville aldrig anvende bolus NMBA<br>(Vælg det præparat du typisk anvender og angiv dosis herfor.)
Angiv dosis<br>__________________________________	
Angiv venligst enhed	◯ g<br>◯ mg<br>◯ µg<br>◯ andet
Uddyb venligst	__________________________________
Angiv dosis	__________________________________
Angiv venligt enhed	◯ g<br>◯ mg<br>◯ µg<br>◯ andet
Uddyb venligst	__________________________________
Angiv dosis	__________________________________
Angiv venligst enhed	◯ g<br>◯ mg<br>◯ µg<br>◯ andet
Uddyb venligst	__________________________________
Angiv dosis	__________________________________
Angiv venligst enhed	◯ g<br>◯ mg<br>◯ µg<br>◯ andet
Uddyb venligst	__________________________________

Hvilket opioid ville du anvende som bolus (single shot, infusion ved høj rate eller kombination) til anæstesi-induktion til en rask ung voksen på 70 kg, 170 cm høj, som skal have foretaget knæartroskopi (eller anden operation som ikke kræver peroperativ eller kirurg-ordineret relaksation?"	◯ Remifentanil<br>◯ Fentanyl<br>◯ Sufentanil<br>◯ Rapifen<br>◯ Jeg ville aldrig anvende bolus opioid uden NMBA i denne case<br>(Vælg det præparat du typisk anvender og angiv dosis herfor.)
Angiv venligst dosis	__________________________________
Angiv venligst enhed	◯ g<br>◯ mg<br>◯ µg<br>◯ andet
Uddyb venligst	__________________________________
Angiv venligst dosis	__________________________________
Angiv venligst enhed	◯ g<br>◯ mg<br>◯ µg<br>◯ andet
Uddyb venligst	__________________________________
Angiv venligst dosis	__________________________________
Angiv venligst enhed	◯ g<br>◯ mg<br>◯ µg<br>◯ andet
Uddyb venligst	__________________________________
Angiv venligst dosis	__________________________________
Angiv venligst enhed	◯ g<br>◯ mg<br>◯ µg<br>◯ andet
Uddyb venligst	__________________________________

Hvilket NMBA ville du anvende som bolus til induktion til en ellers rask ung voksen på 70 kg, 170 cm høj, som skal have foretaget laparoskopisk robotassisteret colonresektion?"	◯ Suxamethonium<br>◯ Rocuronium<br>◯ Cisatracurium<br>◯ Mivacurium<br>◯ Jeg ville aldrig anvende bolus NMBA<br>(Vælg det præparat du typisk anvender og angiv dosis herfor.)
Angiv venligst dosis	__________________________________
Angiv venligst enhed	◯ g<br>◯ mg<br>◯ µg<br>◯ andet
Uddyb venligst	__________________________________
Angiv venligst dosis	__________________________________
Angiv venligst enhed	◯ g<br>◯ mg<br>◯ µg<br>◯ andet
Uddyb venligst	__________________________________
Angiv venligst dosis	__________________________________
Angiv venligst enhed	◯ g<br>◯ mg<br>◯ µg<br>◯ andet
Uddyb venligst	__________________________________
Angiv venligst dosis	__________________________________
Angiv venligst enhed	◯ g<br>◯ mg<br>◯ µg<br>◯ andet
Uddyb venligst	__________________________________

Hvilket opioid ville du anvende som bolus (single shot, infusion ved høj rate eller kombination) til induktion til en ellers rask ung voksen på 70 kg, 170 cm høj, som skal have foretaget laparoskopisk robotassisteret colonresektion?"	◯ Remifentanil<br>◯ Fentanyl<br>◯ Sufentanil<br>◯ Rapifen<br>◯ Jeg ville aldrig anvende bolus opioid uden NMBA i denne case<br>(Vælg det præparat du typisk anvender og angiv dosis herfor.)
Angiv venligst dosis	__________________________________
Angiv venligst enhed	◯ g<br>◯ mg<br>◯ µg<br>◯ andet
Uddyb venligst	__________________________________
Angiv venligst dosis	__________________________________
Angiv venligst enhed	◯ g<br>◯ mg<br>◯ µg<br>◯ andet
Uddyb venligst	__________________________________
Angiv venligst dosis	__________________________________
Angiv venligst enhed	◯ g<br>◯ mg<br>◯ µg<br>◯ andet
Uddyb venligst	__________________________________
Angiv venligst dosis	__________________________________
Angiv venligst enhed	◯ g<br>◯ mg<br>◯ µg<br>◯ andet
Uddyb venligst	__________________________________

Hvilket NMBA ville du give som bolus til induktion til et i øvrigt raskt barn - 3 år og 15 kg - som skal have foretaget tonsillektomi (du har vurderet at barnet skal intuberes).	◯ Suxamethonium<br>◯ Rocuronium<br>◯ Cisatracurium<br>◯ Mivacurium<br>◯ Jeg ville ikke anvende bolus NMBA i denne case<br>(Vælg det præparat du typisk anvender og angiv dosis herfor.)
Angiv venligst dosis	_______________
Angiv venligst enhed	◯ g<br>◯ mg<br>◯ µg<br>◯ andet
Uddyb venligst	_______________
Angiv venligst dosis	_______________
Angiv venligst enhed	◯ g<br>◯ mg<br>◯ µg<br>◯ andet
Uddyb venligst	_______________
Angiv venligst dosis	_______________
Angiv venligst enhed	◯ g<br>◯ mg<br>◯ µg<br>◯ andet
Uddyb venligst	_______________
Angiv venligst dosis	_______________
Angiv venligst enhed	◯ g<br>◯ mg<br>◯ µg<br>◯ andet
Uddyb venligst	_______________

Hvilket opioid ville du anvende som bolus (single shot, infusion ved høj rate eller kombination) til induktion til et i øvrigt raskt barn - 3 år og 15 kg - som skal have foretaget tonsillektomi (du har vurderet at barnet skal intuberes).	◯ Remifentanil<br>◯ Fentanyl<br>◯ Sufentanil<br>◯ Rapifen<br>◯ Jeg ville ikke anvende bolus opioid uden NMBA i denne case<br>(Vælg det præparat du typisk anvender og angiv dosis herfor.)
Angiv venligst dosis	__________________________________
Angiv venligst enhed	◯ g<br>◯ mg<br>◯ µg<br>◯ andet
Uddyb venligst	__________________________________
Angiv venligst dosis	__________________________________
Angiv venligst enhed	◯ g<br>◯ mg<br>◯ µg<br>◯ andet
Uddyb venligst	__________________________________
Angiv venligst dosis	__________________________________
Angiv venligst enhed	◯ g<br>◯ mg<br>◯ µg<br>◯ andet
Uddyb venligst	__________________________________
Angiv venligst dosis	__________________________________
Angiv venligst enhed	◯ g<br>◯ mg<br>◯ µg<br>◯ andet
Uddyb venligst	__________________________________
Er der andet du gerne vil fortælle os om din måde at håndterer ikke-akutte intubationer på?	◯ Ja<br>◯ Nej

Uddyb venligst	__________________________________
Er der noget du gerne vil uddybe eller lade os vide om spørgeskemaet i øvrigt?	◯ Ja<br>◯ Nej
Uddyb venligst	__________________________________

AFSLUTNING

Det var det sidste spørgsmål. Tusinde tak for at du tog dig tiden til at svare. Det er meget værdsat!

Hav en rigtig god dag!
