# Supplementary material for: Neuromuscular Block and Video Laryngoscope to Facilitate Intubation—A Survey of Current Practice in Denmark and Sweden
Source: Acta Anaesthesiol Scand. 2026 Mar 13;70(4):e70200. doi: 10.1111/aas.70200 (PMC12983051; doi:10.1111/aas.70200)
Supplement: Supplementary file 2 — APPENDIX S2: Danish questionnaire for departments. [file AAS-70-0-s002.pdf]

Afdelings niveau

Venligst udfyld nedenstående spørgeskema

Tak!

I dette spørgeskema ønsker vi at opnå viden om den afdeling du er ansat i.

Vi spørger blandt andet til antallet af generelle anæstesier der udføres på jeres afdeling årligt, antallet af speciallæger og hvor mange videolaryngoskoper jeres afdeling råder over. Desuden har vi enkelte spørgsmål til om afdelingen har retningslinjer omhandlende ikke-akut intubation. Det kan være en god ide at have indhentet disse informationer på forhånd. I så fald skulle spørgeskemaet ikke tage mere end ca. 5-10 minutter at besvare.

Den fulde protokol er offentlig tilgængelig via Open Science Framework (https://osf.io/rv7jg) eller kan tilgås på nedenstående link.

[Attachment: "Protocol - Danish national survey on current practice Non-RSI intubation.pdf"]

SAMTYKKEERKLÆRING

Jeg har læst ovenstående beskrivelse af spørgeskemaet.

Jeg er indforstået med, at mine besvarelser er fuldt anonymiserede.

Jeg er også indforstået med, at min samtykke til enhver tid kan tilbagekaldes, hvilket gøres ved at afslutte spørgeskemaet/forlade hjemmesiden uden at indsende svarene

○ Ja, jeg samtykker hermed til deltagelse i studiet
○ Nej, jeg samtykker ikke til deltagelse i studiet

Hvor mange speciallæger i anæstesiologi er ansat i jeres afdeling? __________________________________

Hvor mange operationsstuer har jeres afdelingen i brug på daglig basis? __________________________________

Hvor mange generelle anæstesier udføres der cirka på jeres afdeling om året ? __________________________________

Hvor mange videolaryngoskoper råder jeres afdelinger over? __________________________________

Af hvilket/hvilke mærke(r) er jeres videolarygoskoper?<br><br>(det er muligt at angive mere end én)	☐ Airtraq<br>☐ Ambu King<br>☐ BPL<br>☐ Glidescope<br>☐ HugeMed<br>☐ Karl Storz<br>☐ MedTronic McGrath<br>☐ Niscomed<br>☐ Olympus<br>☐ Pentax<br>☐ secMAC<br>☐ Scope Medical<br>☐ Touren<br>☐ Viscope<br>☐ Andet
Angiv venligst mærket	__________________________________
Foreligger der i jeres afdeling instrukser/vejledninger vedr. valg af medicin til anæstesi induktion ved ikke-akut intubation?	◯ Ja<br>◯ Nej<br>◯ Ved ikke
Anbefaler denne/disse brug af ...	◯ bolus muskelrelaksans<br>◯ bolus opioid (som single shot, infusion ved høj rate eller kombination)<br>◯ både bolus muskelrelaksans og bolus opioid<br>◯ valget er op til anæstesiologen<br>◯ Andet.
Beskriv venligst.	__________________________________
Hvilket muskelrelaksans anbefales specifikt?	◯ Suxamethon<br>◯ Rocuronium<br>◯ Cisatracurium<br>◯ Valget er op til anæstesiologen<br>◯ Andet
Angiv venligst hvilket alternativt muskelrelaksans der er tale om.	__________________________________
Hvilken type opioid anbefales specifikt?	◯ Remifentanil<br>◯ Alfentanil<br>◯ Fentanyl<br>◯ Sufentanil<br>◯ Valget er op til anæstesiologen<br>◯ Andet
Angiv venligst hvilket alternativt opioid der er tale om...	__________________________________

Det var det sidste spørgsmål. Tusinde tak for at du tog dig tiden til at svare. Det er meget værdsat!

Hav en rigtig god dag
