# Supplementary material for: Neuromuscular Block and Video Laryngoscope to Facilitate Intubation—A Survey of Current Practice in Denmark and Sweden
Source: Acta Anaesthesiol Scand. 2026 Mar 13;70(4):e70200. doi: 10.1111/aas.70200 (PMC12983051; doi:10.1111/aas.70200)
Supplement: Supplementary file 3 — APPENDIX S3: English questionnaire for individuals. [file AAS-70-0-s001.pdf]

Individual level

Please complete the questionnaire below.

Thank you!

The following questionnaire forms part of the international research project ROCVIDEO and aims to describe the current approach to non-acute intubations in the operating theatres of public hospitals in Sweden and Denmark. Particular attention is given to the selection of anaesthetic drugs of induction, neuromuscular blocking agents, and the use of video laryngoscopes, as well as key factors influencing these choices.

For a further description please see the attachment (pdf-file) below. The full protocol is publicly available via the Open Science Framework https://osf.io/rv7jg

This research project emanates from CEPRA**.

The questionnaire takes approximately 10 minutes to complete, and your answers are anonymous.

We hope you would like to assist in revealing current clinical practices. Thank you for participating!

Best wishes,
the ROCVIDEO research group

*ROCVIDEO is an independent research project seeking to investigate the benefits and risks of using a fast-acting, high-potency opioid (remifentanil) versus a muscle relaxant (rocuronium) to optimise intubation conditions in patients undergoing videolaryngoscopy-assisted tracheal intubation.

**CEPRA (Collaboration for Evidence-based Practice & Research in Anaesthesia) is a research collaboration aimed to facilitate and support perioperative research programs including large pragmatic multi-centre trials (www.cepra.nu).

[Attachment: "Protocol ROCVIDEO survey.pdf"]

Are you site investigator at your department?

- ◯ Yes
- ◯ No

CONSENT FORM

I have read the description of the questionnaire.

I understand that my answers are fully anonymous

I also understand that my consent can be withdrawn at any time which is done when I stop answering the questionnaire/leave the web site without submitting the answers.

- ◯ Yes, I consent to participate in the study.
- ◯ No, I don´t consent to participate in the study.

PART 1 - DEMOGRAPHICS

Where is your primary place of employment?

- ◯ Public hospital
- ◯ Private hospital/clinic
- ◯ I work at both public and private hospitals/clinics.
- ◯ Prehospital care
- ◯ Other workplace

Please specify other workplace

____

Do you work exclusively in prehospital care?

- ◯ Yes
- ◯ No

Please choose the alternative that gives the best description of your work.

- ◯ I work mainly in the intensive care unit and rarely manage anaesthesia for surgery, treatment or investigative procedures.
- ◯ The main part of my work consists of providing anaesthesia for surgery, treatment or investigative procedures.
- ◯ I work both in the intensive care unit and with providing anaesthesia for surgery, treatment or investigative procedures.
- ◯ Cannot say

What kind of surgery do you frequently provide anaesthesia for? (You may choose several types of surgery)□□

- ☐ Pediatric surgery
- ☐ Obstetric surgery
- ☐ Gynecologic surgery
- ☐ Abdominal surgery
- ☐ Urologic surgery
- ☐ Orthopedic surgery
- ☐ Thoracic surgery
- ☐ Neurosurgery
- ☐ Ear-nose-and-throat surgery
- ☐ Plastic surgery
- ☐ Day surgery
- ☐ Other

If "Other" is chosen; please specify

____

Please state your level of education.

- ◯ Pre-registration house officer (ej legitimerad underläkare)
- ◯ Fully registered medical practitioner (legitimerad läkare)
- ◯ Registrar (ST-läkare)
- ◯ Anaesthesia specialist (specialist i anestesi och intensivvård)

How many years of anaesthesia practice do you have?

- ◯ < 2 years
- ◯ 2-5 years
- ◯ 6-10 years
- ◯ 11-15 years
- ◯ 16-20 years
- ◯ > 20 years

Please state your sex.

- ◯ Male
- ◯ Female
- ◯ Would prefer not to specify

Please enter your age (years).

____

In which country is your primary place of employment?

- ◯ The Faroe Islands
- ◯ Greenland
- ◯ Denmark
- ◯ Sweden

In what part of the country is your primary place of employment?<br><br>(Please choose one part only)	◯ Norra Sverige (Jämtland, Norrbotten, Västerbotten och Västernorrland)<br>◯ Mellan-Sverige (Dalarna, Gävleborg, Södermanland, Uppsala, Västmanland, Värmland, Örebro)<br>◯ Stockholm-Gotland<br>◯ Västra Sverige (Halland, Västra Götaland)<br>◯ Sydöstra Sverige (Jönköping, Kalmar, Östergötland)<br>◯ Södra Sverige (Blekinge, Kronoberg, Skåne)
In what region in Norra Sverige do you primarily work? (Please choose one region)	◯ Region Jämtland Härjedalen, Östersunds sjukhus<br>◯ Region Norrbotten<br>◯ Region Västerbotten<br>◯ Region Västernorrland
In which hospital in Region Norrbotten do you primarily work? (Please choose only one)	◯ Piteå Älvdals sjukhus<br>◯ Sunderby sjukhus<br>◯ Kalix sjukhus<br>◯ Kiruna sjukhus<br>◯ Gällivare sjukhus
In which hospital in Region Västerbotten do you primarily work? (Please choose one hospital only)	◯ Norrlands Universitetssjukhus<br>◯ Lasarettet Skellefteå<br>◯ Lasarettet Lycksele
In which hospital in Region Västernorrland do you primarily work? (Please choose only one)	◯ Länssjukhuset Sundsvall och Härnösand<br>◯ Sollefteå sjukhus<br>◯ Örnsköldsviks sjukhus
In what region in Mellan-Sverige do you primarily work? (Please choose only one region)	◯ Region Dalarna<br>◯ Region Gävleborg<br>◯ Region Södermanland<br>◯ Region Uppsala<br>◯ Region Västmanland<br>◯ Region Värmland<br>◯ Region Örebro län
In which hospital in Region Dalarna do you primarily work? (Please choose one hospital only)	◯ Falu lasarett<br>◯ Lasarettet i Mora
In which hospital in Region Gävleborg do you primarily work? (Please choose one hospital only)	◯ Gävle sjukhus<br>◯ Hudiksvalls sjukhus
In which hospital in Region Södermanland do you primarily work? (Please choose one hospital only)	◯ Mälarsjukhuset<br>◯ Kullbergska sjukhuset<br>◯ Nyköpings lasarett
In which hospital in Region Uppsala do you primarily work? (Please choose one hospital only)	◯ Akademiska sjukhuset<br>◯ Lasarettet i Enköping
In which hospital in Region Västmanland do you primarily work? (Please choose one hospital only)	◯ Västmanlands sjukhus Västerås<br>◯ Västmanlands sjukhus Köping
In which hospital in Region Värmland do you primarily work? (Please choose one hospital only)	◯ Centralsjukhuset Karlstad<br>◯ Sjukhuset Arvika<br>◯ Sjukhuset Torsby

In which hospital in Region Örebro län do you primarily work? (Please choose one hospital only)	◯ Universitetssjukhuset Örebro<br>◯ Karlskoga lasarett<br>◯ Lindesberg lasarett
In which hospital in Region Stockholm-Gotland is your primary place of employment?<br>(Please choose one hospital only)	◯ Astrid Lindgrens Barnsjukhus<br>◯ Capio S:t Görans sjukhus<br>◯ Danderyds sjukhus<br>◯ Ersta Diakoni<br>◯ Karolinska Universitetssjukhuset Huddinge<br>◯ Karolinska Universitetssjukhuset Solna<br>◯ Karolinska Universitetssjukhuset Thorax<br>◯ Norrtälje sjukhus<br>◯ S:t Eriks ögonsjukhus<br>◯ Södersjukhuset<br>◯ Södertälje sjukhus<br>◯ Visby lasarett
In what region in Västra Sverige is your primary place of employment? (Please choose only one region)	◯ Region Halland<br>◯ Region Västra Götaland
In which hospital in Region Halland is your primary place of employment?<br>(Please choose one hospital only)	◯ Hallands sjukhus Halmstad<br>◯ Hallands sjukhus Varberg<br>◯ Kungsbacka sjukhus
In which public hospital in Region Västra Götaland is your primary place of employment?<br>(Please choose one hospital only)	◯ Alingsås lasarett<br>◯ Drottning Silvias Barnsjukhus<br>◯ Kungälvs sjukhus<br>◯ Norra Älvsborgs sjukhus<br>◯ Södra Älvsborgs Sjukhus Borås.<br>◯ Sahlgrenska sjukhuset<br>◯ Sahlgrenska, Mölndals sjukhus<br>◯ Sahlgrenska, Östra sjukhuset<br>◯ Skaraborgs sjukhus Lidköping<br>◯ Skaraborgs sjukhus Skövde
In what region in Sydöstra Sverige is your primary place of employment? (Please choose only one region)	◯ Region Jönköping län<br>◯ Region Kalmar län<br>◯ Region Östergötland
In which public hospital in Region Jönköping län is your primary place of employment?<br>(Please choose one hospital only)	◯ Länssjukhuset Ryhov<br>◯ Höglandssjukhuset Eksjö<br>◯ Sjukhuset Värnamo
In which hospital in Region Kalmar län is your primary place of employment?<br>(Please choose one hospital only)	◯ Länssjukhuset Kalmar<br>◯ Oskarshamns sjukhus<br>◯ Sjukhuset Västervik
In which hospital in Region Östergötland is your primary place of employment?<br>(Please choose one hospital only)	◯ Lasarettet i Motala<br>◯ Universitetssjukhuset i Linköping<br>◯ Vrinnevisjukhuset
In what region in Södra Sverige is your primary place of employment? (Please choose only one region)	◯ Region Blekinge, Blekingesjukhuset<br>◯ Region Kronoberg<br>◯ Region Skåne
In which hospital in Region Kronoberg is your primary place of employment?<br>(Please choose one hospital only)	◯ Centrallasarettet i Växjö<br>◯ Lasarettet i Ljungby

In which hospital in Region Skåne is your primary place of employment?
(Please choose one hospital only)

- ◯ Skånes Universitetssjukhus Lund
- ◯ Skånes Universitetssjukhus Malmö
- ◯ Centralsjukhuset Kristianstad
- ◯ Helsingborgs lasarett
- ◯ Lasarettet Ystad
- ◯ Lasarettet Trelleborg
- ◯ Lasarettet i Landskrona
- ◯ Ängelholms sjukhus
- ◯ Hässleholms sjukhus

PART 2 - VIDEOLARYNGOSCOPY

In this section we would like information about your use of videolaryngoscopes for NON-ACUTE intubations.

Does your department have access to videolaryngoscopes? ▯▯

- ◯ Yes, in every operation theatre (i varje operationssal)
- ◯ Yes, videolaryngoscopes are easily accessible - but not in every operation theatre
- ◯ No, I don´t have easy access to a videolaryngoscope
- ◯ No, I don´t have access to a videolaryngoscope at all
- ◯ Other

If "Other" is chosen please describe shortly.

____

Which option best describes to what extent you use a video laryngoscope for non-acute intubations? (Please choose only one option)

- ◯ I always use a video laryngoscope (approximately 100%)
- ◯ I frequently use a video laryngoscope (approximately 75%)
- ◯ I use a traditional laryngoscope and a video laryngoscope with equal frequency (approximately 50%)
- ◯ I rarely use a video laryngoscope (approximately 25%)
- ◯ I hardly ever use a video laryngoscope (approximately 0 %)
- ◯ I don´t know

Which option best describes why you choose the videolaryngoscope for non-acute intubations?

- ◯ I use a video laryngoscope when indicated (if the airway has been assessed as difficult, the mobility of the neck is reduced, severe obesity etc)
- ◯ I use the video laryngoscope for educational purposes
- ◯ I use the video laryngoscope because it is the only laryngoscope available
- ◯ I prefer to always use the video laryngoscope
- ◯ Other reason

(Only one option is possible to choose)

If "Other reason" is chosen; please specify.

____

PART 3 - DRUGS FOR INDUCTION & INTUBATION

We would like information about what drugs you prefer to use for intubation. All questions refer to NON-ACUTE intubations.

PLEASE NOTE: a bolus opioid can either be given as a "single shot" or by briefly running an infusion at a high rate or a combination of these two.

In non-acute intubations how often do you use a bolus of neuromuscular blocking agent (NMBA) versus a bolus of opioid (single shot, high rate infusion or a combination of these two) without NMBA to facilitate intubation?	◯ I mainly use NMBA<br>◯ I most often use an opioid without NMBA<br>◯ Other
If "Other" is chosen please describe briefly the method used.	__________________________________
Which NMBA would you typically use for a non-acute intubation?	◯ Suxamethonium<br>◯ Rocuronium<br>◯ Cisatracurium<br>◯ Other<br>(Please indicate the one you choose most often)
If "Other" is chosen please specify which NMBA.	__________________________________
Which opioid would you typically use as a bolus dose if you planned NOT to administer a bolus dose of NMBA to facilitate a non-acute endotracheal intubation? (Please indicate the one you choose most often)	◯ Remifentanil<br>◯ Alfentanil<br>◯ Fentanyl<br>◯ Sufentanil<br>◯ I never/almost never use an opioid without NMBA for intubation<br>◯ Other
If "Other" is chosen; please specify which drug.	__________________________________
When using opioids without NMBA for intubation, how often do you need to add a NMBA to achieve successful intubation?	◯ in approximately 0-24% of all intubations<br>◯ in approximately 25-49% of all intubations<br>◯ in approximately 50-74% of all intubations<br>◯ in approximately 75-100% of all intubations<br>◯ I never use opioids without NMBA for intubation
Please indicate which statement best fits with: "I use a bolus dose NMBA because..."	◯ I adhere to the guideline of our department<br>◯ I adhere to national/European/international guidelines<br>◯ We have a tradition of doing so in our department<br>◯ Because NMBA provides the best conditions for successful intubation<br>◯ Other
If "Other" is chosen; please describe shortly.	__________________________________

Which NMBA would you give as a bolus dose to facilitate tracheal intubation for a healthy young adult, with a height of 170 cm and weight of 70 kg, undergoing an elective knee arthroscopy (or other type of procedure that doesn´t require muscle relaxation for surgical reasons)?	◯ Suxamethonium<br>◯ Rocuronium<br>◯ Cisatracurium<br>◯ Mivacurium<br>◯ I wouldn´t use a bolus dose of NMBA<br>◯ Other<br>(Choose the drug and the dose you normally would use)
If "Other" is chosen; please specify which NMBA, including the dose and unit.	__________________________________
Please specify the dose (without decimals).	__________________________________<br>(Please change unit if you want to state dose with decimals)
Please specify the unit<br>◯ g ◯ mg ◯ µg ◯ other	
Please specify the dose (without decimals).	__________________________________<br>(Please change unit if you want to state dose with decimals)
Please specify the unit.	◯ g<br>◯ mg<br>◯ µg<br>◯ other
Please specify the dose (without decimals).	__________________________________<br>(Please change unit if you want to state dose with decimals)
Please specify the unit.	◯ g<br>◯ mg<br>◯ µg<br>◯ other
Please specify the dose (without decimals).	__________________________________<br>(Please change unit if you want to state dose with decimals)
Please specify the unit.	◯ g<br>◯ mg<br>◯ µg<br>◯ other

Which opioid would you give as a bolus dose (single shot, high rate infusion or a combination of these two) during anaesthesia induction to facilitate tracheal intubation for a healthy young adult, with a height of 170 cm and weight of 70 kg, undergoing an elective knee arthroscopy (or other type of procedure that doesn´t require muscle relaxation for surgical reasons) if you planned NOT to use a NMBA?	◯ Remifentanil<br>◯ Fentanyl<br>◯ Sufentanil<br>◯ Alfentanil<br>◯ I would never use a bolus dose of opioid without NMBA in such a case<br>◯ Other<br>(Choose the drug and the dose you normally would use)
If "Other" is chosen; please specify which opioid, including dose and unit, you would use.	______________________
Please specify the dose (without decimals).	______________________<br>(Please change unit if you want to state dose with decimals)
Please specify the unit.	◯ g<br>◯ mg<br>◯ µg<br>◯ other
	______________________
Please specify the dose (without decimals).	______________________<br>(Please change unit if you want to state dose with decimals)
Please specify the unit.	◯ g<br>◯ mg<br>◯ µg<br>◯ other
Please specify the dose (without decimals).	______________________<br>(Please change unit if you want to state dose with decimals)
Please specify the unit.	◯ g<br>◯ mg<br>◯ µg<br>◯ other
Please specify the dose (without decimals).	______________________<br>(Please change unit if you want to state dose with decimals)
Please specify the unit.	◯ g<br>◯ mg<br>◯ µg<br>◯ other

Which NMBA would you give as a bolus dose during anaesthesia induction for a healthy young adult, with a height of 170 cm and weight of 70 kg, undergoing a laparoscopic robot-assisted colon resection?	◯ Suxamethonium<br>◯ Rocuronium<br>◯ Cisatracurium<br>◯ Mivacurium<br>◯ I wouldn´t use a bolus dose of NMBA<br>◯ other<br>(Cchoose the drug and the dose you normally would use)
If "Other" is chosen; please specify which NMBA, including the dose and unit you would use.	__________________________________
Please specify the dose (without decimals).	__________________________________<br>(Please change unit if you want to state dose with decimals)
Please specify the unit.	◯ g<br>◯ mg<br>◯ µg<br>◯ other
Please specify the dose (without decimals).	__________________________________<br>(Please change unit if you want to state dose with decimals)
Please specify the unit.	◯ g<br>◯ mg<br>◯ µg<br>◯ other
Please specify the dose (without decimals).	__________________________________<br>(Please change unit if you want to state dose with decimals)
Please specify the unit.	◯ g<br>◯ mg<br>◯ µg<br>◯ other
Please specify the dose (without decimals).	__________________________________<br>(Please change unit if you want to state dose with decimals)
Please specify the unit.	◯ g<br>◯ mg<br>◯ µg<br>◯ other

Which opioid would you give as a bolus dose (single shot, high rate infusion or a combination of these two) during anaesthesia induction for a healthy young adult, with a height of 170 cm and weight of 70 kg, undergoing a laparoscopic robot-assisted colon resection if you planned NOT to use a NMBA?	◯ Remifentanil<br>◯ Fentanyl<br>◯ Sufentanil<br>◯ Alfentanil<br>◯ I would never use a bolus dose of opioid without NMBA in this case<br>◯ other<br>(Choose the drug and the dose you normally would use)
If "Other" is chosen: please specify which opioid, including the dose and unit you would use.	__________________________
Please specify the dose (without decimals).	__________________________<br>(Please change unit if you want to state dose with decimals)
Please specify the unit.	◯ g<br>◯ mg<br>◯ µg<br>◯ other
Please specify the dose (without decimals).	__________________________<br>(Please change unit if you want to state dose with decimals)
Please specify the unit.	◯ g<br>◯ mg<br>◯ µg<br>◯ other
Please specify the dose (without decimals).	__________________________<br>(Please change unit if you want to state dose with decimals)
Please specify the unit.	◯ g<br>◯ mg<br>◯ µg<br>◯ other
Please specify the dose (without decimals).	__________________________<br>(Please change unit if you want to state dose with decimals)
Please specify the unit.	◯ g<br>◯ mg<br>◯ µg<br>◯ other

Which NMBA would you give as a bolus dose during anaesthesia induction for a healthy 3-year old child with a weight of 15 kg, requiring tracheal intubation for a tonsillectomy?	◯ Suxamethonium<br>◯ Rocuronium<br>◯ Cisatracurium<br>◯ Mivacurium<br>◯ I wouldn´t use a NMBA for intubation<br>◯ other<br>(Choose the drug and the dose you normally would use))
If "Other" is chosen; please specify which NMBA, including the dose and unit you would use.	______________________
Please specify the dose (without decimals).	______________________<br>(Please change unit if you want to state dose with decimals)
Please specify the unit.	◯ g<br>◯ mg<br>◯ µg<br>◯ other
Please specify the dose (without decimals).	______________________<br>(Please change unit if you want to state dose with decimals)
Please specify the unit.	◯ g<br>◯ mg<br>◯ µg<br>◯ other
Please specify the dose (without decimals).	______________________<br>(Please change unit if you want to state dose with decimals)
Please specify the unit.	◯ g<br>◯ mg<br>◯ µg<br>◯ other
Please specify the dose (without decimals).	______________________<br>(Please change unit if you want to state dose with decimals)
Please specify the unit.	◯ g<br>◯ mg<br>◯ µg<br>◯ other

Which opioid would you give as a bolus dose (single shot, high rate infusion or a combination of these two) during anaesthesia induction for a healthy 3-year child with a weight of 15 kg, undergoing tonsillectomy if you planned NOT to use a NMBA? (You have assessed that the child needs intubating.)	◯ Remifentanil<br>◯ Fentanyl<br>◯ Sufentanil<br>◯ Alfentanil<br>◯ I wouldn´t use a bolus dose of opioid without NMBA in this case<br>◯ other<br>((Choose the drug and the dose you normally would use))
If "Other" is chosen please specify which opioid, including the dose and unit you would use.	__________________________________
Please specify the dose (without decimals).	__________________________________<br>(Please change unit if you want to state dose with decimals)
Please specify the unit.	◯ g<br>◯ mg<br>◯ µg<br>◯ other
Please specify the dose (without decimals).	__________________________________<br>(Please change unit if you want to state dose with decimals)
Please specify the unit.	◯ g<br>◯ mg<br>◯ µg<br>◯ other
Please specify the dose (without decimals).	__________________________________<br>(Please change unit if you want to state dose with decimals)
Please specify the unit.	◯ g<br>◯ mg<br>◯ µg<br>◯ other
Please specify the dose (without decimals).	__________________________________<br>(Please change unit if you want to state dose with decimals)
Please specify the unit.	◯ g<br>◯ mg<br>◯ µg<br>◯ other
Is there anything else you would like to tell us about your way of handling non-emergency intubations?	◯ Yes<br>◯ No
Please describe.	__________________________________

Is there anything else about the questionnaire you would like to tell us?

◯ Yes
◯ No

Please describe.

____

CONCLUSION

That was the last question of the questionnaire. THANK YOU for taking the time to participate!
