# Supplementary material for: Neuromuscular Block and Video Laryngoscope to Facilitate Intubation—A Survey of Current Practice in Denmark and Sweden
Source: Acta Anaesthesiol Scand. 2026 Mar 13;70(4):e70200. doi: 10.1111/aas.70200 (PMC12983051; doi:10.1111/aas.70200)
Supplement: Supplementary file 4 — APPENDIX S4: English questionnaire for departments. [file AAS-70-0-s005.pdf]

Department level

Please complete the questionnaire below.

Thank you!

This part of the survey regards the number of general anaesthetics performed in your department annually, the number of specialist anaesthetists and how many videolaryngoscopes that are available at your department. It also includes questions regarding guidelines for non-acute intubation at your department.

The full protocol is publicly available at the Open Science Framework: https://osf.io/rv7jg

[Attachment: "Protocol ROCVIDEO survey.pdf"]

STATEMENT OF CONSENT

I have read the description of the questionnaire.

The answers will be handled confidentially and data presented in a way that individual answers cannot be identified.

I understand that my consent can be withdrawn at any time which is done when I stop answering the questionnaire/leave the web site without submitting the answers.

- ◯ Yes, I consent to participating in the study
- ◯ No, I don´t consent to participate in the study

How many specialist anaesthetists are employed at your department? (Not including anaesthetists in training.)

____

How many operation theatres (operationssalar) are at use on a daily basis at your department?

____

How many general anaesthesias are performed annually at your department?

____

How many video laryngoscopes are available at your department?

____

What brand/brands of video laryngoscope do you have at your department? (more than one can be

chosen)

- ☐ Airtraq
- ☐ Ambu King
- ☐ BPL
- ☐ Glidescope
- ☐ HugeMed
- ☐ Karl Storz
- ☐ MedTronic McGrath
- ☐ Niscomed
- ☐ Olympus
- ☐ Pentax
- ☐ secMAC
- ☐ Scope Medical
- ☐ Touren
- ☐ Viscope
- ☐ other

If "Other" brand of videolaryngoscope is chosen, please specify what brand/brands.

____

In your department; do you have guidelines regarding the choice of drugs for anaesthesia induction in non-acute intubation?	◯ Yes<br>◯ No<br>◯ I don´t know
If guidelines exist; do they recommend the use of	◯ a bolus dose of neuromuscular blocking agent (NMBA)<br>◯ a bolus dose of opioid<br>◯ both a bolus dose of opioid and NMBA<br>◯ the choice is left to the anaesthetist<br>◯ other
If "Other" is chosen; please describe.	__________________________________
Which NMBA is recommended specifically?□□	◯ suxamethonium<br>◯ rocuronium<br>◯ cisatracurium<br>◯ the choice is left to the anaesthetist<br>◯ other
If "Other" is chosen; please specify which NMBA.	__________________________________
Which opioid is recommended specifically?	◯ remifentanil<br>◯ alfentanil<br>◯ fentanyl<br>◯ sufentanil<br>◯ the choice is left to the anaesthetist<br>◯ other
If "Other" opioid is chosen; please describe.	__________________________________

This was the last question of the survey. The ROCVIDEO research group thanks you for taking the time to participate thus adding to our knowledge in this field. Your effort is much appreciated and of great value!
