# Supplementary material for: Neuromuscular Block and Video Laryngoscope to Facilitate Intubation—A Survey of Current Practice in Denmark and Sweden
Source: Acta Anaesthesiol Scand. 2026 Mar 13;70(4):e70200. doi: 10.1111/aas.70200 (PMC12983051; doi:10.1111/aas.70200)
Supplement: Supplementary file 5 — APPENDIX S5: Region of employment. [file AAS-70-0-s006.docx]

**Supplement 5.** Region of employment of respondents of the survey

|  | | | |
| --- | --- | --- | --- |
|  | **Denmark (N=700)** | **Sweden (N=1071)** | **Overall (N=1771)** |
| **Region** |  |  |  |
| Hovedstaden | 229 (32.7%) | 0 (0%) | 229 (12.9%) |
| Midtjylland | 160 (22.9%) | 0 (0%) | 160 (9.0%) |
| Nordjylland | 69 (9.9%) | 0 (0%) | 69 (3.9%) |
| Sjaelland | 92 (13.1%) | 0 (0%) | 92 (5.2%) |
| Syddanmark | 150 (21.4%) | 0 (0%) | 150 (8.5%) |
| Mellan Sverige | 0 (0%) | 234 (21.8%) | 234 (13.2%) |
| Norra Sverige | 0 (0%) | 108 (10.1%) | 108 (6.1%) |
| Södra Sverige | 0 (0%) | 175 (16.3%) | 175 (9.9%) |
| Stockholm-Gotland | 0 (0%) | 266 (24.8%) | 266 (15.0%) |
| Sydöstra Sverige | 0 (0%) | 118 (11.0%) | 118 (6.7%) |
| Västra Sverige | 0 (0%) | 170 (15.9%) | 170 (9.6%) |
