# Supplementary figures and images for: Neuromuscular Block and Video Laryngoscope to Facilitate Intubation—A Survey of Current Practice in Denmark and Sweden
Source: Acta Anaesthesiol Scand. 2026 Mar 13;70(4):e70200. doi: 10.1111/aas.70200 (PMC12983051; doi:10.1111/aas.70200)

**Supplement 6**. Percentage of respondents providing anaesthesia for various types of surgery.


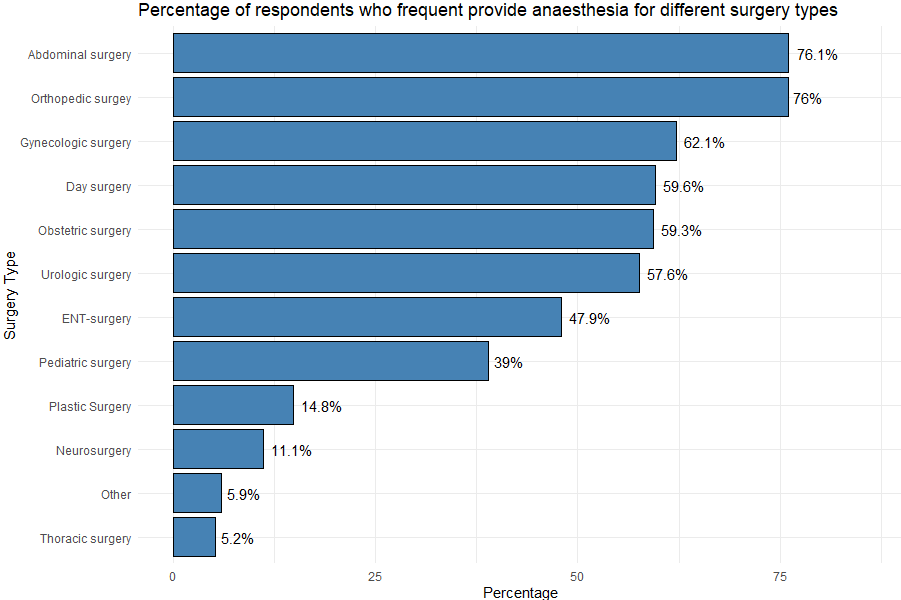

Supplement: Supplementary file 6 — APPENDIX S6: Types of surgery. [file AAS-70-0-s003.docx]
